# Supplementary material for: Sex modulation of faces prediction error in the autistic brain
Source: Commun Biol. 2024 Jan 25;7:127. doi: 10.1038/s42003-024-05807-4 (PMC10810845; doi:10.1038/s42003-024-05807-4)
Supplement: Supplementary file 2 — Supplementary Information (new) [file 42003_2024_5807_MOESM2_ESM.pdf]

# Supplementary Material to Sex modulation of faces prediction error in the autistic brain.

Adeline Lacroix[1], Sylvain Harquel[1,2], Martial Mermillod[1], Marta Garrido[3,4], Leonardo Barbosa[1,5], Laurent Vercueil[1], David Aleysson[1], Frédéric Dutheil[6], Klara Kovarski[7,8], & Marie Gomot[9]

[1] Univ. Grenoble Alpes, Univ. Savoie Mont Blanc, CNRS, LPNC, 38000 Grenoble, France.

[2] Defitech Chair in Clinical Neuroengineering, Center for Neuroprosthetics and Brain Mind Institute, EPFL, Geneva, Switzerland.

[3] Cognitive Neuroscience and Computational Psychiatry Lab, Melbourne School of Psychological Sciences, The University of Melbourne, Australia.

[4] Australian Research Council Centre of Excellence for Integrative Brain Function, Australia.

[5] Fralin Biomedical Research Institute at VTC, Virginia Tech, Roanoke, VA 24016, USA.

[6] Université Clermont Auvergne, CNRS, LaPSCo, CHU Clermont-Ferrand, WittyFit, F-63000 Clermont-Ferrand, France.

[7] Sorbonne Université, Faculté des Lettres, INSPE, Paris, France.

[8] LaPsyDÉ, Université Paris-Cité, CNRS, Paris, France.

[9] UMR 1253 iBrain, Université de Tours, Inserm, Tours, France.

Author note

Adeline Lacroix ORCID ID: https://orcid.org/0000-0001-6990-5949

Correspondence concerning this article should be addressed to Adeline Lacroix, Laboratoire de Psychologie et NeuroCognition, Univ. Grenoble Alpes, 1251 avenue centrale, Grenoble, France. E-mail: adeline.lacroix@univ-grenoble-alpes.fr

Supplementary Material to Sex modulation of faces prediction error in the autistic brain.

# Material and methods

## Participants

All autistic participants were clinically diagnosed prior to the study by an experienced clinical team, according to the criteria of the Diagnostic and Statistical Manual of Mental Disorders, DSM-IV or DSM-5 [1,2]. Standardized tools were used for diagnosis such as the Autism Diagnostic Observation Schedule[3], the Autism Diagnostic Interview-Revised[4] and/or the Asperger Syndrome Diagnostic Interview[5]. Thirteen participants provided their diagnostic reports without additional diagnostic instruments.

# Results

## Behavioral results

*Supplementary Table 1: Analyses of group and sex differences for the detection of the colored face*

| Effect | df | MSE | F | pes | p.value |
|---|---|---|---|---|---|
| Group | 1, 85 | 0.59 | 0.59 | .007 | .443 |
| Sex | 1, 85 | 0.59 | 1.30 | .015 | .258 |
| Group:Sex | 1, 85 | 0.59 | 0.27 | .003 | .606 |

Results did not show significant difference on behavioral performances between group, sex and their interaction (all $p > .25$). The mean $d'$ value for colored face detection was high (Mean $d' = 4.56 \pm 0.77$).

**Posterior analyses MMN1 amplitudes**

*Supplementary Table 2: Analysis of amplitudes on posterior electrodes in the first time window.*

| Effect | df | MSE | F | pes | p.value |
|---|---|---|---|---|---|
| Group | 1, 85 | 3.01 | 0.04 | <.001 | .833 |
| Sex | 1, 85 | 3.01 | 1.82 | .021 | .181 |
| Group:Sex | 1, 85 | 3.01 | 3.99 * | .045 | .049 |
| SF | 1, 85 | 1.00 | 62.72 *** | .425 | <.001 |
| Group:SF | 1, 85 | 1.00 | 0.55 | .006 | .461 |
| Sex:SF | 1, 85 | 1.00 | 8.09 ** | .087 | .006 |
| Group:Sex:SF | 1, 85 | 1.00 | 0.42 | .005 | .520 |
| laterality | 1, 85 | 0.83 | 18.85 *** | .182 | <.001 |
| Group:laterality | 1, 85 | 0.83 | 4.76 * | .053 | .032 |
| Sex:laterality | 1, 85 | 0.83 | 0.29 | .003 | .590 |
| Group:Sex:laterality | 1, 85 | 0.83 | 2.25 | .026 | .137 |
| SF:laterality | 1, 85 | 0.40 | 0.09 | .001 | .759 |
| Group:SF:laterality | 1, 85 | 0.40 | 0.58 | .007 | .448 |
| Sex:SF:laterality | 1, 85 | 0.40 | 0.18 | .002 | .675 |
| Group:Sex:SF:laterality | 1, 85 | 0.40 | 0.02 | <.001 | .892 |

In addition to the results reported in the main text related to our hypotheses, the results showed that the right hemisphere had a more negative MMR than the left hemisphere, qualified by an interaction between Group and Hemisphere. Post-hoc tests revealed a significantly smaller MMR in the left compared to the right hemisphere in ASD ($\beta = 0.63$, $p < 0.001$) but not in TD. This does not change the interpretation of the results in the main text.

**Posterior analyses MMN1 latencies**

*Supplementary Table 3: Analysis of latencies on posterior electrodes in the first time window.*

| Effect | df | MSE | F | pes | p.value |
|---|---|---|---|---|---|
| Group | 1, 85 | 926.87 | 0.47 | .005 | .495 |
| Sex | 1, 85 | 926.87 | 3.65 + | .041 | .059 |
| Group:Sex | 1, 85 | 926.87 | 0.50 | .006 | .481 |
| SF | 1, 85 | 288.88 | 5.89 * | .065 | .017 |
| Group:SF | 1, 85 | 288.88 | 3.79 + | .043 | .055 |
| Sex:SF | 1, 85 | 288.88 | 0.23 | .003 | .633 |
| Group:Sex:SF | 1, 85 | 288.88 | 4.08 * | .046 | .047 |
| laterality | 1, 85 | 321.46 | 0.11 | .001 | .736 |
| Group:laterality | 1, 85 | 321.46 | 2.00 | .023 | .161 |
| Sex:laterality | 1, 85 | 321.46 | 0.00 | <.001 | .946 |
| Group:Sex:laterality | 1, 85 | 321.46 | 0.06 | <.001 | .800 |
| SF:laterality | 1, 85 | 230.20 | 0.03 | <.001 | .858 |
| Group:SF:laterality | 1, 85 | 230.20 | 2.83 + | .032 | .096 |
| Sex:SF:laterality | 1, 85 | 230.20 | 0.06 | <.001 | .802 |
| Group:Sex:SF:laterality | 1, 85 | 230.20 | 0.03 | <.001 | .855 |

**Posterior analyses MMN2 Mean Amplitudes**

*Supplementary Table 4: Analysis of the mean amplitude on posterior electrodes in the second time window.*

| Effect | df | MSE | F | pes | p.value |
|---|---|---|---|---|---|
| Group | 1, 85 | 1.78 | 0.00 | <.001 | .975 |
| Sex | 1, 85 | 1.78 | 0.17 | .002 | .681 |

| | | | | | |
|---|---|---|---|---|---|
| Group:Sex | 1, 85 | 1.78 | 0.03 | <.001 | .865 |
| SF | 1, 85 | 0.71 | 40.19 *** | .321 | <.001 |
| Group:SF | 1, 85 | 0.71 | 2.37 | .027 | .127 |
| Sex:SF | 1, 85 | 0.71 | 0.05 | <.001 | .832 |
| Group:Sex:SF | 1, 85 | 0.71 | 0.06 | <.001 | .810 |
| laterality | 1, 85 | 0.86 | 1.14 | .013 | .289 |
| Group:laterality | 1, 85 | 0.86 | 1.48 | .017 | .227 |
| Sex:laterality | 1, 85 | 0.86 | 1.62 | .019 | .207 |
| Group:Sex:laterality | 1, 85 | 0.86 | 1.37 | .016 | .244 |
| SF:laterality | 1, 85 | 0.36 | 0.30 | .004 | .584 |
| Group:SF:laterality | 1, 85 | 0.36 | 0.35 | .004 | .557 |
| Sex:SF:laterality | 1, 85 | 0.36 | 0.03 | <.001 | .856 |
| Group:Sex:SF:laterality | 1, 85 | 0.36 | 0.16 | .002 | .691 |

**Central analyses MMN1 amplitudes**

*Supplementary Table 5: Analysis of amplitudes on central electrodes in the first time window.*

| **Effect** | **df** | **MSE** | **F** | **pes** | **p.value** |
|---|---|---|---|---|---|
| Group | 1, 85 | 1.37 | 3.04 + | .035 | .085 |
| Sex | 1, 85 | 1.37 | 1.68 | .019 | .198 |
| Group:Sex | 1, 85 | 1.37 | 2.78 + | .032 | .099 |
| SF | 1, 85 | 0.65 | 41.88 *** | .330 | <.001 |
| Group:SF | 1, 85 | 0.65 | 0.68 | .008 | .413 |
| Sex:SF | 1, 85 | 0.65 | 4.87 * | .054 | .030 |
| Group:Sex:SF | 1, 85 | 0.65 | 0.00 | <.001 | .968 |

**Central analyses MMN1 latencies**

*Supplementary Table 6: Analysis of latencies on central electrodes in the first time window.*

| Effect | df | MSE | F | pes | p.value |
|---|---|---|---|---|---|
| Group | 1, 85 | 639.77 | 3.76 + | .042 | .056 |
| Sex | 1, 85 | 639.77 | 1.99 | .023 | .162 |
| Group:Sex | 1, 85 | 639.77 | 0.12 | .001 | .734 |
| SF | 1, 85 | 420.01 | 1.13 | .013 | .291 |
| Group:SF | 1, 85 | 420.01 | 2.52 | .029 | .116 |
| Sex:SF | 1, 85 | 420.01 | 0.90 | .010 | .345 |
| Group:Sex:SF | 1, 85 | 420.01 | 0.03 | <.001 | .858 |

**Central analyses MMN2 mean amplitudes**

*Supplementary Table 7: Analysis of mean amplitude on central electrodes in the second time window.*

| Effect | df | MSE | F | pes | p.value |
|---|---|---|---|---|---|
| Group | 1, 85 | 1.31 | 3.11 + | .035 | .082 |
| Sex | 1, 85 | 1.31 | 0.47 | .005 | .495 |
| Group:Sex | 1, 85 | 1.31 | 0.69 | .008 | .407 |
| SF | 1, 85 | 0.56 | 58.17 *** | .406 | <.001 |
| Group:SF | 1, 85 | 0.56 | 2.87 + | .033 | .094 |
| Sex:SF | 1, 85 | 0.56 | 2.60 | .030 | .110 |
| Group:Sex:SF | 1, 85 | 0.56 | 0.04 | <.001 | .850 |

**P100 Amplitudes**

*Supplementary Table 8: Analysis of P100 amplitudes.*

| Effect | df | MSE | F | pes | p.value |
|---|---|---|---|---|---|
| Group | 1, 85 | 36.06 | 0.63 | .007 | .429 |
| Sex | 1, 85 | 36.06 | 8.88 ** | .095 | .004 |
| Group:Sex | 1, 85 | 36.06 | 3.09 + | .035 | .082 |
| SF | 1.39, 118.34 | 2.75 | 40.30 *** | .322 | <.001 |
| Group:SF | 1.39, 118.34 | 2.75 | 2.52 | .029 | .103 |
| Sex:SF | 1.39, 118.34 | 2.75 | 0.63 | .007 | .481 |
| Group:Sex:SF | 1.39, 118.34 | 2.75 | 0.93 | .011 | .366 |
| channel | 1, 85 | 10.78 | 11.21 ** | .116 | .001 |
| Group:channel | 1, 85 | 10.78 | 1.88 | .022 | .174 |
| Sex:channel | 1, 85 | 10.78 | 1.58 | .018 | .213 |
| Group:Sex:channel | 1, 85 | 10.78 | 0.74 | .009 | .391 |
| SF:channel | 1.28, 108.82 | 2.12 | 0.76 | .009 | .416 |
| Group:SF:channel | 1.28, 108.82 | 2.12 | 1.11 | .013 | .310 |
| Sex:SF:channel | 1.28, 108.82 | 2.12 | 1.52 | .018 | .224 |
| Group:Sex:SF:channel | 1.28, 108.82 | 2.12 | 0.23 | .003 | .689 |

The amplitude on PO8 was larger than on PO7 ($F$ (1, 85) = 11.21, $p$ = 0.001, $\eta^2$ = 0.12) revealing a lateralization of the response.

**P100 Latency**

*Supplementary Table 9: Analysis of P100 latencies.*

| Effect | df | MSE | F | pes | p.value |
|---|---|---|---|---|---|
| Group | 1, 85 | 623.14 | 0.66 | .008 | .418 |
| Sex | 1, 85 | 623.14 | 0.79 | .009 | .376 |
| Group:Sex | 1, 85 | 623.14 | 0.81 | .009 | .371 |
| SF | 1.53, 130.36 | 254.05 | 36.36 *** | .300 | <.001 |
| Group:SF | 1.53, 130.36 | 254.05 | 1.87 | .022 | .168 |
| Sex:SF | 1.53, 130.36 | 254.05 | 0.34 | .004 | .658 |
| Group:Sex:SF | 1.53, 130.36 | 254.05 | 1.62 | .019 | .207 |
| channel | 1, 85 | 245.52 | 3.11 + | .035 | .082 |
| Group:channel | 1, 85 | 245.52 | 0.08 | <.001 | .785 |
| Sex:channel | 1, 85 | 245.52 | 0.63 | .007 | .429 |
| Group:Sex:channel | 1, 85 | 245.52 | 0.10 | .001 | .747 |
| SF:channel | 1.64, 139.77 | 123.17 | 0.10 | .001 | .867 |
| Group:SF:channel | 1.64, 139.77 | 123.17 | 0.93 | .011 | .382 |
| Sex:SF:channel | 1.64, 139.77 | 123.17 | 3.06 + | .035 | .060 |
| Group:Sex:SF:channel | 1.64, 139.77 | 123.17 | 0.60 | .007 | .519 |

**N170 Amplitude**

*Supplementary Table 10: Analysis of N170 amplitudes.*

| Effect | df | MSE | F | pes | p.value |
|---|---|---|---|---|---|
| Group | 1, 85 | 13.81 | 1.96 | .023 | .165 |
| Sex | 1, 85 | 13.81 | 1.45 | .017 | .233 |
| Group:Sex | 1, 85 | 13.81 | 0.44 | .005 | .510 |
| SF | 1.29, 109.51 | 2.03 | 47.05 *** | .356 | <.001 |

| | | | | | |
|---|---|---|---|---|---|
| Group:SF | 1.29, 109.51 | 2.03 | 3.08 + | .035 | .072 |
| Sex:SF | 1.29, 109.51 | 2.03 | 2.04 | .023 | .151 |
| Group:Sex:SF | 1.29, 109.51 | 2.03 | 0.16 | .002 | .754 |
| channel | 1, 85 | 4.98 | 1.32 | .015 | .254 |
| Group:channel | 1, 85 | 4.98 | 3.90 + | .044 | .052 |
| Sex:channel | 1, 85 | 4.98 | 0.53 | .006 | .471 |
| Group:Sex:channel | 1, 85 | 4.98 | 3.59 + | .041 | .061 |
| SF:channel | 1.60, 135.92 | 1.21 | 0.17 | .002 | .795 |
| Group:SF:channel | 1.60, 135.92 | 1.21 | 0.07 | <.001 | .893 |
| Sex:SF:channel | 1.60, 135.92 | 1.21 | 0.21 | .002 | .764 |
| Group:Sex:SF:channel | 1.60, 135.92 | 1.21 | 1.91 | .022 | .161 |

## N170 Latency

*Supplementary Table 11: Analysis of N170 latencies.*

| Effect | df | MSE | F | pes | p.value |
|---|---|---|---|---|---|
| Group | 1, 85 | 611.99 | 0.10 | .001 | .749 |
| Sex | 1, 85 | 611.99 | 6.06 * | .067 | .016 |
| Group:Sex | 1, 85 | 611.99 | 0.11 | .001 | .738 |
| SF | 1.97, 167.07 | 86.00 | 23.64 *** | .218 | <.001 |
| Group:SF | 1.97, 167.07 | 86.00 | 0.04 | <.001 | .957 |
| Sex:SF | 1.97, 167.07 | 86.00 | 1.76 | .020 | .177 |
| Group:Sex:SF | 1.97, 167.07 | 86.00 | 1.19 | .014 | .307 |
| channel | 1, 85 | 177.70 | 4.48 * | .050 | .037 |
| Group:channel | 1, 85 | 177.70 | 1.04 | .012 | .310 |
| Sex:channel | 1, 85 | 177.70 | 0.23 | .003 | .634 |
| Group:Sex:channel | 1, 85 | 177.70 | 0.15 | .002 | .696 |

| | | | | | |
|---|---|---|---|---|---|
| SF:channel | 1.93, 164.37 | 68.60 | 0.54 | .006 | .578 |
| Group:SF:channel | 1.93, 164.37 | 68.60 | 1.61 | .019 | .205 |
| Sex:SF:channel | 1.93, 164.37 | 68.60 | 1.40 | .016 | .249 |
| Group:Sex:SF:channel | 1.93, 164.37 | 68.60 | 0.93 | .011 | .393 |

There was a significant main effect of Hemisphere ($F$ (1, 85) = 4.48, $p$ = 0.037, $\eta^2$ = 0.05), P8 presenting faster latencies than P7.

**Males only**

All analyses on MMR, P100 and N170 were also performed on males only in order to be able to compare the results with other studies, that included a majority of men. In these analyses, we found no Group effect or interactions between Group and other factors, except for the P100, for which we found a main effect of SF ($F$ (2, 68) = 23.80, $p$ = <0.001, $\eta^2$ = 0.36), a main effect of Group ($F$ (1, 43) = 7.35, $p$ = 0.010, $\eta^2$ = 0.15), qualified by an interaction between Group and SF ($F$ (2, 68) = 4.17, $p$ = 0.028, $\eta^2$ = 0.09). Post-hoc tests confirmed that P100 amplitude was larger for BSF and LSF than for HSF in TD (all $p$ < .001) and that the difference between conditions was not significant in ASD (all $p$ >.1) but also revealed a decreased P100 amplitude in ASD males compared to TD males in BSF ($\beta$ = -1.72, $p$ = 0.023), which was marginally significant in LSF ($\beta$ = -1.51, $p$ = 0.065) and not significant in HSF ($\beta$ = -0.76, $p$ = 0.708).

**Correlation**

Pearson correlation was used to analyze the correlation between age, education, Autism Spectrum Quotient, Full Scale IQ, Performance IQ, Verbal IQ, and diagnostic age with the electrophysiological response. The electrophysiological response of interest was the difference between HSF MMR and LSF MMR. P-values are corrected for multiple inference according to Holm’s method. Significant correlations are colored in the following matrices.

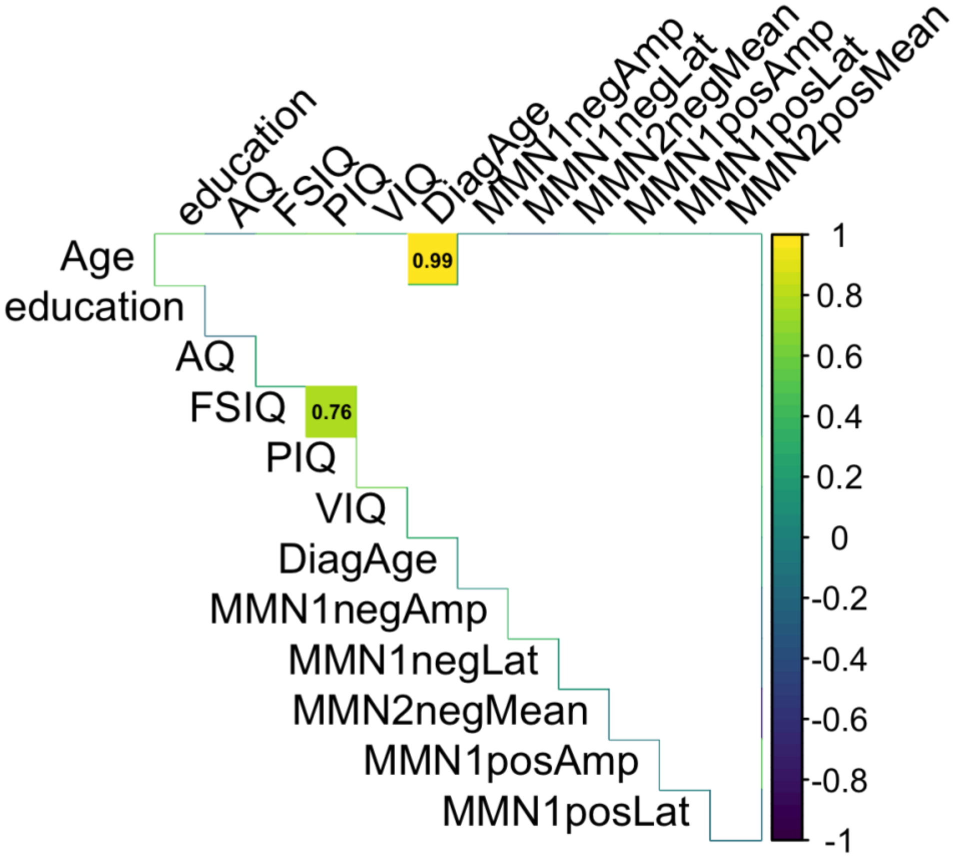


Supplementary Figure 1: Correlation analyses in autistic females.

While no significant correlation is observed in autistic females, in autistic males we observed that higher PIQ (which is the non-verbal IQ, is correlated with a smaller difference between HSF MMR and LSF MMR in the first positive peak of the MMR. Additionally, older age is associated with higher AQ scores in autistic males.

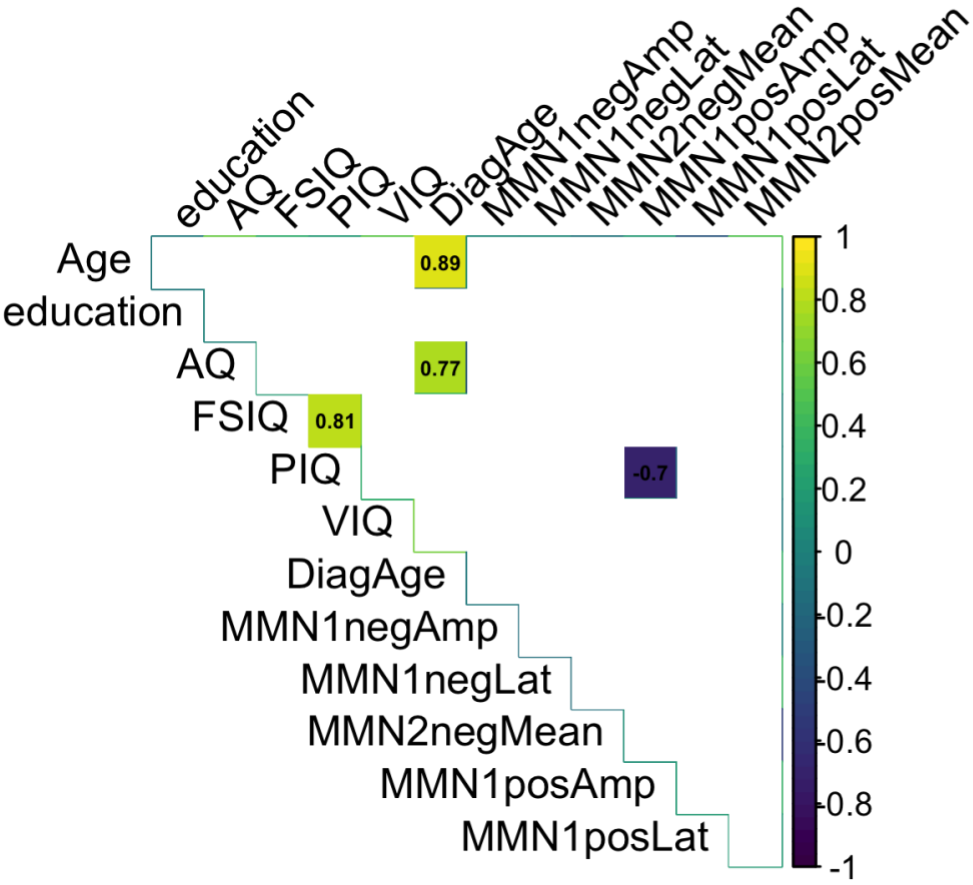


Supplementary Figure 2: Correlation analyses in autistic males.

Finally, the positive and negative components of the difference between HSF MMR and LSF MMR are correlated in non-autistic males and in non-autistic females. This is not observed in autism, confirming some specificities of autistic males and females.

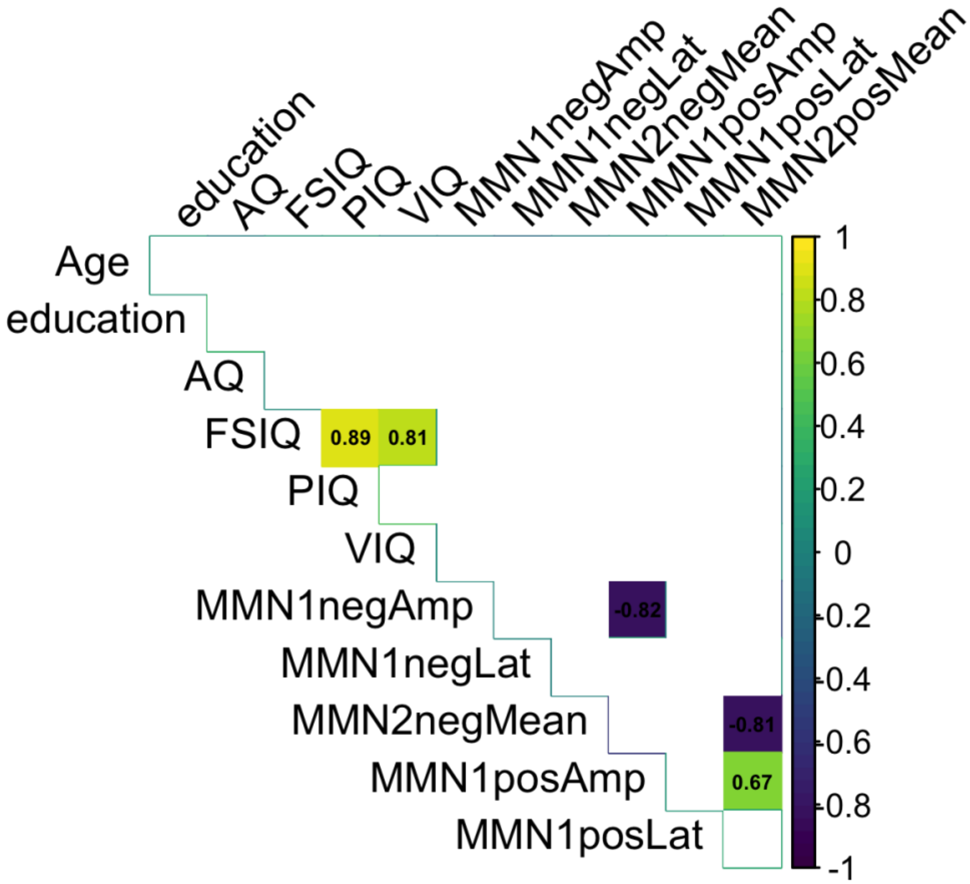


Supplementary Figure 3: Correlation analyses in non autistic females.

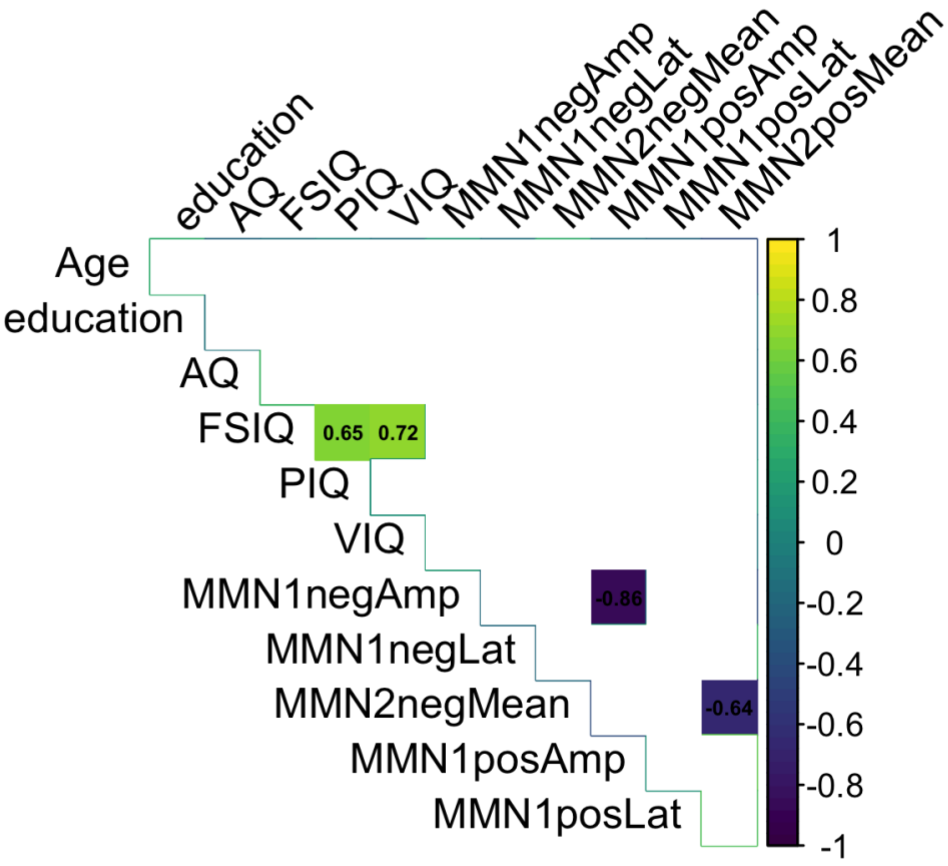


Supplementary Figure 4: Correlation analyses in non autistic males.

**Supplementary References**

1. American Psychiatric Association. Diagnostic and Statistical Manual of Mental Disorders, –Text Revision (DSM-IV-TR). American Psychiatric Association. *Washington, DC* (2000).

2. American Psychiatric Association. *Diagnostic and statistical manual of mental disorders (DSM-5®)*. (American Psychiatric Pub, 2013).

3. Lord, C. *et al.* Austism diagnostic observation schedule: A standardized observation of communicative and social behavior. *J Autism Dev Disord* **19**, 185–212 (1989).

4. Lord, C., Rutter, M. & Couteur, A. L. Autism Diagnostic Interview-Revised: A revised version of a diagnostic interview for caregivers of individuals with possible pervasive developmental disorders. *J Autism Dev Disord* **24**, 659–685 (1994).

5. Gillberg, C., Gillberg, C., Råstam, M. & Wentz, E. The Asperger Syndrome (and High-Functioning Autism) Diagnostic Interview (ASDI): A Preliminary Study of a New Structured Clinical Interview. *Autism* **5**, 57–66 (2001).
