# Supplementary material for: Sex modulation of faces prediction error in the autistic brain
Source: Commun Biol. 2024 Jan 25;7:127. doi: 10.1038/s42003-024-05807-4 (PMC10810845; doi:10.1038/s42003-024-05807-4)
Supplement: Supplementary file 3 — Reporting summary [file 42003_2024_5807_MOESM3_ESM.pdf]

nature portfolio

Corresponding author(s): Adeline Lacroix

Last updated by author(s): 18/12/23

Reporting Summary

Nature Portfolio wishes to improve the reproducibility of the work that we publish. This form provides structure for consistency and transparency in reporting. For further information on Nature Portfolio policies, see our Editorial Policies and the Editorial Policy Checklist.

Statistics

For all statistical analyses, confirm that the following items are present in the figure legend, table legend, main text, or Methods section.

n/a	Confirmed	
☐	☒	The exact sample size (*n*) for each experimental group/condition, given as a discrete number and unit of measurement
☐	☒	A statement on whether measurements were taken from distinct samples or whether the same sample was measured repeatedly
☐	☒	The statistical test(s) used AND whether they are one- or two-sided<br>*Only common tests should be described solely by name; describe more complex techniques in the Methods section.*
☐	☒	A description of all covariates tested
☐	☒	A description of any assumptions or corrections, such as tests of normality and adjustment for multiple comparisons
☐	☒	A full description of the statistical parameters including central tendency (e.g. means) or other basic estimates (e.g. regression coefficient) AND variation (e.g. standard deviation) or associated estimates of uncertainty (e.g. confidence intervals)
☐	☒	For null hypothesis testing, the test statistic (e.g. *F*, *t*, *r*) with confidence intervals, effect sizes, degrees of freedom and *P* value noted<br>*Give P values as exact values whenever suitable.*
☒	☐	For Bayesian analysis, information on the choice of priors and Markov chain Monte Carlo settings
☒	☐	For hierarchical and complex designs, identification of the appropriate level for tests and full reporting of outcomes
☐	☒	Estimates of effect sizes (e.g. Cohen's *d*, Pearson's *r*), indicating how they were calculated

Our web collection on statistics for biologists contains articles on many of the points above.

Software and code

Policy information about availability of computer code

Data collection	Stimuli were displayed using Presentation® software (Neurobehavioral Systems, 451 Inc., Berkeley, CA, www.neurobs.com).<br>BrainAmp amplifiers and EasyCaps (Brain Products GmbH, Germany) with 96 active electrodes following the 10-5 standard system were used for EEG recording.
Data analysis	Brainstorm software and MATLAB (The MathWorks Inc.) scripts were used for EEG pre-processing.<br>R 4.2.3 and R studio 2023.06.0+421 were used to analyse the data (ANOVA) (R scripts are available on the OSF repository).

For manuscripts utilizing custom algorithms or software that are central to the research but not yet described in published literature, software must be made available to editors and reviewers. We strongly encourage code deposition in a community repository (e.g. GitHub). See the Nature Portfolio guidelines for submitting code & software for further information.

Data

Policy information about availability of data

All manuscripts must include a data availability statement. This statement should provide the following information, where applicable:

- Accession codes, unique identifiers, or web links for publicly available datasets
- A description of any restrictions on data availability
- For clinical datasets or third party data, please ensure that the statement adheres to our policy

The ERP data, scripts, non-identiying demographical data, and materials used in this study are openly available on the Open Science Framework (OSF) at https://osf.io/yd6h7/?view_only=bcd4c3f36cc74b22b2e26f6bb91ad7ce. Raw data are available on request. Should anything be missing, we are more than willing to provide it upon request.

Research involving human participants, their data, or biological material

Policy information about studies with human participants or human data. See also policy information about sex, gender (identity/presentation), and sexual orientation and race, ethnicity and racism.

Reporting on sex and gender	The sex of the participant (observed at birth) was one of the main variable of interest in our study and was taken into account in our analyses. Thus, we paid attention to have a similar number of males and females in each group.
Reporting on race, ethnicity, or other socially relevant groupings	We did not reported race or ethnicity as it was not relevant for the study.
Population characteristics	We collected age and IQ in order to match autistic and non autistic participants in order to control these variables.
Recruitment	Autistic participants were recruited with the help of the local expertise center for the autism diagnosis, which is specialized in diagnosing autism in adulthood, creating a bias. Autistic participants were also recruited by local associations and by mailing list. Non autistic participants were recruited via personnal and social networks, and with mailing list of volunteers. The recruitment done by various channels allow to reduce selection bias, even if this bias can not totally be ruled out.
Ethics oversight	Comité de Protection des Personnes Ile de France 1—IRB/IORG: IORG0009918)

Note that full information on the approval of the study protocol must also be provided in the manuscript.

Field-specific reporting

Please select the one below that is the best fit for your research. If you are not sure, read the appropriate sections before making your selection.

☐ Life sciences ☒ Behavioural & social sciences ☐ Ecological, evolutionary & environmental sciences

For a reference copy of the document with all sections, see nature.com/documents/nr-reporting-summary-flat.pdf

Behavioural & social sciences study design

All studies must disclose on these points even when the disclosure is negative.

Study description	quantitative experimental study
Research sample	20 autistic females (Mean Age = 31± 9), 21 autistic males (Mean Age = 28 ±9), 24 non autistic females (mean age = 31 ± 9) and non-autistc males (Mean age = 29 ± 5). The groups are matched in age and IQ.
Sampling strategy	Since this is the first study investigating sex differences in the electrophysiological response of autistic adults, there were no similar studies available for reference. Therefore, the sample size was determined heuristically, based on EEG studies conducted with autistic participants, typically involving around 40 participants (20 autistic). To investigate sex differences, the sample size was doubled (80 participants). To take into account participant with possible bad signal that should be discarded and to have a slightly larger sample size, we upsize the sample of 25 % resulting in the final recruitment of 97 participants.
Data collection	BrainAmp amplifiers and EasyCaps (Brain Products GmbH, Germany) with 96 active electrodes following the 10-5 standard system were used for EEG recording, during which impedance was kept below 25 kΩ. A sampling rate of 1,000 Hz was used for signal recording, with an anti-aliasing filter at 500 Hz. The ground electrode for EEG was FPz, while the reference was FCz. Two electrodes on the left and right outer canthi of the eyes and two others above and below the left eye were used to record the horizontal and vertical electrooculographic (EOG) activity (hEOG and vEOG) respectively. The ground electrode for EOG was positioned on the left base of the neck.<br>Stimuli were displayed centrally using Presentation® software (Neurobehavioral Systems, Inc., Berkeley, CA, www.neurobs.com) on a CRT screen of 37 × 29.6 cm with a refresh rate of 75 Hz and a resolution of 1,280 × 1,024 pixels. They were presented at a viewing distance of 87 cm. Participants were instructed to look at the fixation cross and to detect the colored face by pressing a button as

	quickly as possible. Then, they were left alone in the recording room and were monitored with a camera during the 30 minutes recording session.
Timing	EEG acquisitions were performed at the IRMaGe neurophysiology facility (Grenoble, France) from September 2020 to July 2021.
Data exclusions	97 participants were recruited but two autistic males were removed due to bad vision (logMAR > 0.2). Six additionnal participants (2 autistic females, one autistic male, and 3 NA females) were removed due to bad signal after visual inspection, resulting in a final sample of 89 participants.<br>For each participant, muscular artifacts were manually discarded. The signal was then re-referenced using average reference. Eye movements were corrected using signal-space projection (SSP). Finally, a band-pass filter of 0.1 - 40 Hz was applied to the cleaned signal and trials were epoched from 100 ms pre-stimulus to 600 ms post-stimulus, except for the first three trials of the sequences and trials presented after deviants or target, which were excluded. In the end, 1 % of the trials of NA participants and 2.3 % of the trials of ASD participants were discarded during pre-processing. Next, bad channels were interpolated based on neighboring channels. A mean of 4 channels was interpolated per participant. For six participants, the signal was recorded on 64 electrodes only and missing electrodes, distributed on the scalp, were also interpolated for statistical analyses.<br>Source reconstruction was performed for each participant in each condition, except for one autistic participant, who had an atypical signal in frontal areas.
Non-participation	no participant dropped out
Randomization	participants were in the autistic or in the non autistc group, based on their diagnosis.

Reporting for specific materials, systems and methods

We require information from authors about some types of materials, experimental systems and methods used in many studies. Here, indicate whether each material, system or method listed is relevant to your study. If you are not sure if a list item applies to your research, read the appropriate section before selecting a response.

Materials & experimental systems

n/a	Involved in the study	
☒	☐	Antibodies
☒	☐	Eukaryotic cell lines
☒	☐	Palaeontology and archaeology
☒	☐	Animals and other organisms
☒	☐	Clinical data
☒	☐	Dual use research of concern
☒	☐	Plants

Methods

n/a	Involved in the study	
☒	☐	ChIP-seq
☒	☐	Flow cytometry
☒	☐	MRI-based neuroimaging
